# A practical test of the link between perceived identifiability and prosociality with two field studies

**DOI:** 10.1038/s41598-022-17248-2

**Published:** 2022-07-31

**Authors:** Yashvin Seetahul, Tobias Greitemeyer

**Affiliations:** University of Innsbruck, Innsbruck, Austria

**Keywords:** Psychology, Human behaviour

## Abstract

Covering the face with masks in public settings has been recommended since the start of the pandemic. Because faces provide information about identity, and that face masks hide a portion of the face, it is plausible to expect individuals who wear a mask to consider themselves less identifiable. Prior research suggests that perceived identifiability is positively related to prosocial behavior, and with two pre-registered field studies (total N = 5706) we provide a currently relevant and practical test of this relation. Our findings indicate that mask wearers and non-wearers display equivalent levels of helping behavior (Studies 1 and 2), although mask wearers have a lower level of perceived identifiability than those without a mask (Study 2). Overall, our findings suggest that claims that face masks are related to selfish behavior are not warranted, and that there is no practical link between perceived identifiability and prosocial behavior.

## Introduction

Several real-world examples suggest that reduced identifiability is related to anti-normative behaviors. For instance, individuals are known to write harsh, sometimes offensive, comments online when it is possible to remain hidden behind unidentifiable usernames^1^. Festivals that allow individuals to remain surrounded by crowds are also often accompanied by several mild to severe incidents^2–5^. When conducting more extreme anti-normative behaviors, anonymity may even be a necessary condition, in which case individuals may hide their faces for instrumental reasons. For instance, robbers often wear a balaclava when committing a heist, and hackers are known to wear a mask of a historical figure when making their demands. It is plausible that the possibility to reduce one’s identifiability is positively linked to anti-normative behaviors. In fact, situations that reduce the identifiability of individuals, such as when individuals’ faces are hidden with sanitary face masks, may facilitate the occurrence of such behaviors.

Faces provide information about age, gender, emotional states, mental states and, more broadly, identity^6,7^. In fact, throughout evolution, individuals have evolved to use faces as the primary cue to identify one another (see Allen^8^). It, therefore, makes sense that if major parts of the face are hidden, individuals will consider themselves less identifiable. This reduced perceived identifiability would subsequently facilitate behaviors that are anti-normative. Similarly to how hiding parts of the face may be linked to increased antisocial behaviors, it may also be linked to reduced prosocial behaviors. For instance, individuals who had their eyes hidden with sunglasses behave more selfishly than those wearing clear glasses^9^. It is then plausible to expect that other facial accessories that hide large areas of the face would lead to similar observations. With two pre-registered field studies, we investigate whether individuals wearing a sanitary face mask during the COVID-19 pandemic behave less prosocially than individuals without a face mask.

## Costs of helping and refusing to help

Prosocial behaviors refer to a general category of voluntary behaviors that are carried out with the intent of providing benefits to others. They may benefit other individuals, entire groups, or society as a whole^10^. It comes as no surprise that “[a]ssisting others, donating to charity, cooperating with others, and intervening to save another person’s life are all acts that societies generally value”^11^. However, while these behaviors will—by definition—provide benefits to the recipient, they come at a cost to the benefactor (e.g., time, effort, money). Although prosocial behaviors can also provide material, social, psychological, or even physical gains^12–15^ to the benefactor, if perceived costs outweigh the benefits people will typically decide not to provide help (for reviews, see Batson, Ahmad and Stocks^16^, Cialdini and Goldstein^17^, and Rahal and Fiedler^18^).

Importantly, while behaving prosocially may come at personal costs, refusing to do so is not without its own toll. Not helping someone in need may lead to social reproach. Because selfishness is not valued by social norms^19^, behaving selfishly may lead to public censure and ostracism^20^. Given that “human beings have a pervasive drive to form and maintain at least a minimum quantity of lasting, positive, and significant interpersonal relationships”^21^, they will be highly motivated to avoid the risk of being disliked or rejected^22^. The risk of social reproach is all the more relevant when refusing to comply to an explicit and direct request for help, in which case “many people agree to things—even things they would prefer not to do—simply to avoid the considerable discomfort of saying ‘no.’”^23^. In fact, individuals may prefer avoiding the social interaction altogether, rather than having to refuse helping someone (e.g., Andreoni, Rao, Trachtman^24^, Dana, Cain and Dawes^25^, and DellaVigna, List, and Malmender^26^).

Hence, when deciding to help, individuals not only consider costs and benefits of helping but also the costs of refusing. If individuals perceive the costs of refusing as being higher, they may (unhappily) comply with the request. Here, we focus on a situation that may reduce the risk of social reproach, and thus make refusing to help easier: when individuals are less identifiable.

## The effect of reduced (perceived) identifiability

Understanding the situational conditions in which individuals would behave less prosocially has been a topic of interest for researchers in social psychology for over a century. For instance, in 1895 Le Bon 1895 suggested that when individuals are in a crowd, they would be more inclined to behave anti-normatively. According to him, “becoming submerged in a throng leads individuals to lose both external and internal constraints upon their behaviour” (see^27^). Festinger, Pepitone, and Newcomb^28^ referred to this phenomenon as “deindividuation” and described it as a state in which individuals provide less effort to behave normatively because they are not seen or paid attention to as individuals (p. 382). Zimbardo^29^ further stressed that this phenomenon would occur in any situation that reduce self-observation, self-evaluation, and concern for social evaluation. He proposed that in typical social situations, the anticipation of emotions such as guilt, shame, or fear would motivate individuals to make the effort to conduct normative behavior and inhibit anti-normative ones (see Nickerson^30^). Deindividuation would occur in situations that would reduce the anticipatory guilt, shame, or fear. In 1982, Prentice-Dunn and Rogers^31^ proposed to classify the situational predictors that induce a deindividuation state and reduce normative behavior as situations that would shift the individuals own attention from themselves to other individuals, groups, or objects (i.e., “attentional cues”) or situations that would reduce the concern for evaluation (i.e., “accountability cues”). One such circumstance is when individuals are less identifiable. Reduced identifiability would reduce the long-term negative consequences of anti-normative behavior (i.e., others are less likely to make social reproach in the long term if they do not know who the anti-normative individual is), and as such, individuals who perceive themselves as being less identifiable should have less concern for social evaluation. Although researchers often refer to this situation as being anonymous (i.e., completely unidentifiable), in practice the decrease of identifiability may occur in various degrees.

A focus on anonymity as a predictor of the deindividuated state started early on, with Le Bon suggesting that “[b]eing indistinguishable from others in the crowd leads individuals to lose all sense of individuality and hence the sense of individual responsibility that normally controls behaviour” (see Reicher et al.^27^, p. 192). Several researchers found empirical evidence that low (perceived) identifiability would induce a deindividuated state. Singer, Brush, and Lublin^32^ manipulated anonymity in the lab by giving half their participants oversized lab coats that made them indistinguishable from one another, and found that participants in the lowered identifiability condition used more “obscene” language than participants in the control condition. Similarly, Zimbardo^29^ manipulated anonymity with oversized lab coats and hoods and asked participants to shock confederates (similar to a Milgram paradigm). Those in the low identifiability condition shocked confederates more than those in the control condition. In 1976, Diener, Fraser, Beaman, and Kelem^33^ conducted a study on 1039 children trick-or-treating with costumes and masks during Halloween. Children were provided the opportunity to steal candies, and the authors manipulated identifiability by asking half of the children to give their names and address. They found that 10.5% of identifiable children stole candies, while 80% of those who remained anonymous did so. And in a similar study with children during Halloween, Miller and Rowold^34^ counted children who stole candies and compared those who wore a mask and those who did not. They found that 62% of children wearing a mask stole candies, while only 37% of those not wearing one did the same.

Despite these early findings, several other findings found no evidence of the deindividuation state^35,36^ and a meta-analysis conducted by Postmes and Spears^37^ of sixty empirical studies showed little support for the existence of the deindividuation phenomenon. Furthermore, early studies focused more on anti-normative behaviors in the form of increased antisocial behaviors and showed little evidence that it would lead to decreased prosocial behaviors. However, more recent studies have suggested the possibility that reduced perceived identifiability may indeed lead to a deindividuated state. Mullen, Migdal, and Rozell^38^ found that masks lead to a decrease in self-awareness and saliency of social identity. As a consequence, reduced perceived identifiability has been found to decrease prosocial behavior. For instance, individuals made 84% lower donations when their behavior was not observed and their names hidden, compared to when they were identifiable^39^, and the subjective belief that their behavior could be clearly identified increased individuals’ contribution behavior^40,41^. Similarly, Zhong et al.^8,9^ found that participants wearing sunglasses had an illusory sense of anonymity and behaved more selfishly than those wearing clear glasses.

## The present research

The years 2020, 2021 and 2022 have been severely affected by the COVID-19 outbreak, and covering the face with masks is recommended and oftentimes mandatory in public settings to reduce the risk of transmission of the virus. With the possibility that face masks could remain highly present in social contexts in post-COVID-19 years, the health, social and psychological consequences of face masks have been topics of interest in public debates and in research (e.g.^42–49^).

Because these masks cover the mouth and nose area, they hide a large portion of the face and may reduce the actual and perceived identifiability, which then may decrease the extent to which individuals feel concerned by social evaluation. Consequently, face masks should decrease prosocial behaviors. The unusual circumstances of the COVID-19 pandemic, thus, allow us to conduct a currently relevant and practical test of the theoretical relation between perceived identifiability and prosocial behaviors. In two pre-registered field studies, we approached individuals in public areas who were either wearing a face mask or not wearing one. These individuals were provided the opportunity to behave prosocially to various degrees by answering questions in a long survey. Verbal consent was obtained from all individuals who agreed to answer the survey. The first page of the survey informed them that the participation was voluntary and that they could stop at any time (which was also reminded on all pages of the survey). We also counted how many individuals refused to answer the survey in each group (Mask groups vs. No Mask group). In both studies, we compared the number of questions answered by individuals wearing a face mask and individuals not wearing one. Study 2 also examined the impact of wearing a face mask on the participant’s level of perceived identifiability. Both studies conform to the guidelines of the of the Austrian sanitary regulations in place during the data collection, the Declaration of Helsinki and the good scientific practice and recommendations of the European Union for Social Science and Humanities. According to the Ethics Committee of the University of Innsbruck, no ethical approval was required for this study as no identifiable human data was gathered and no intervention or manipulation took place in the context of this study. All data generated and analyzed during these studies as we followed the mentioned guidelines, as well as registrations, materials, and code are available on the OSF repository: https://doi.org/10.17605/OSF.IO/CRZM3.

## Study 1

### Results

#### Descriptive statistics

From our N = 398 participants, 186 wore a mask during the entire interaction (i.e., Mask group), 212 did not wear a mask during the entire interaction (i.e., No Mask group).

**Figure 1:**
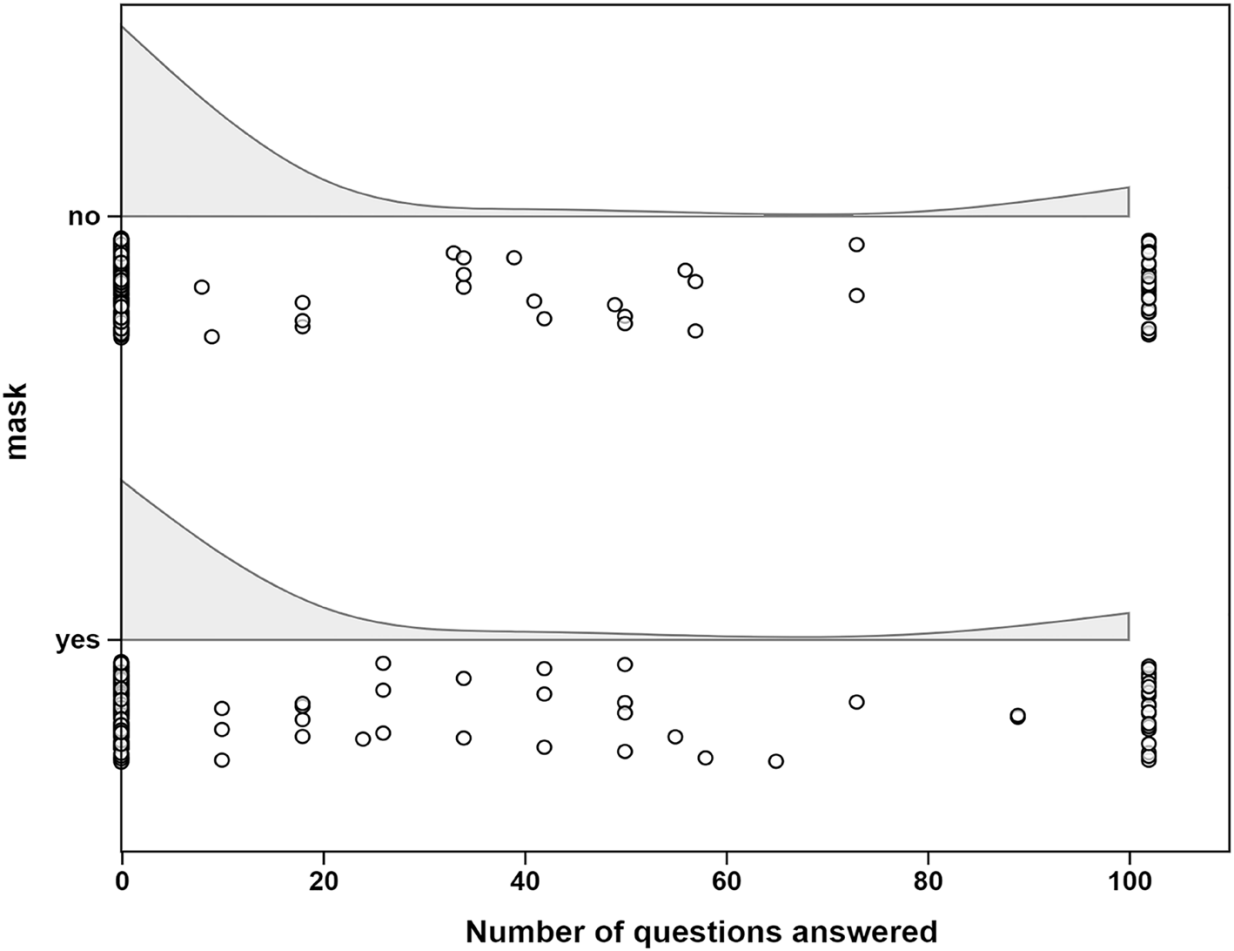
Raincloud plots of prosocial measure scores for the two groups.

The overall mean for the prosocial behavior measure was 17.39 with a standard deviation of 35.2, a minimum of 0 and maximum of 102. The skewness of the distribution is 1.78 and the kurtosis is 4.44. Most participants in both groups answered 0 questions (see Fig. 1). The prosocial measure for the Mask group was M = 18.68 (SD = 35.89) and for the No Mask group was M = 16.25 (SD = 34.62).

#### Hypothesis test

As per our pre-registration (https://doi.org/10.17605/OSF.IO/UMY4F), we conducted a one-sided Student’s t-test on R to test whether the number of questions answered by participants in the Mask group was smaller than in the No Mask group. Given the continuous ratio nature of the dependent variable, and two-level categorical nature of the independent variable, the one-sided Student’s t-test provides more power than alternative analytic strategies to observe whether the Mask group has a smaller mean than the No Mask group.

As indicated by the skewness and kurtosis of our measure in the descriptive statistics, the distribution of our measure violates the normal distribution resulting in a strong “floor effect” and a moderate “ceiling effect”. Transforming the distribution did not allow us to obtain a normal distribution, whether the transformation was Log (skewness = 1.31, kurtosis = 2.84), Square-Root (skewness = 1.53, kurtosis = 3.6) or Reciprocal (skewness = 1.78, kurtosis = 4.44). However, while normality of distribution of the dependent variable is necessary for small samples, non-normal distributions do not affect the validity of t-tests for large samples^50^, hence the non-normal distribution of our prosocial measure is unlikely to impact the validity of our conclusions.

The result of our t-test indicates that the difference between the two groups was non-significant, *t*(396) = − 0.69, *p* = 0.754 (one-tailed), d = − 0.07, 95% CI [− Infinity, 8.267]. As the descriptive statistics indicate, participants in the Mask group actually answered more questions than participants in the No Mask group, which is the opposite of our hypothesis.

#### Additional test

We conducted an exploratory one-sided Student’s t-test to see if we could potentially make the inference that face masks increase prosocial behavior, but the difference was non-significant, *t*(396) = − 0.69, *p* = 0.246 (one-tailed), d = 0.07, 95% CI [− 3.4, Infinity].

**Figure 2:**
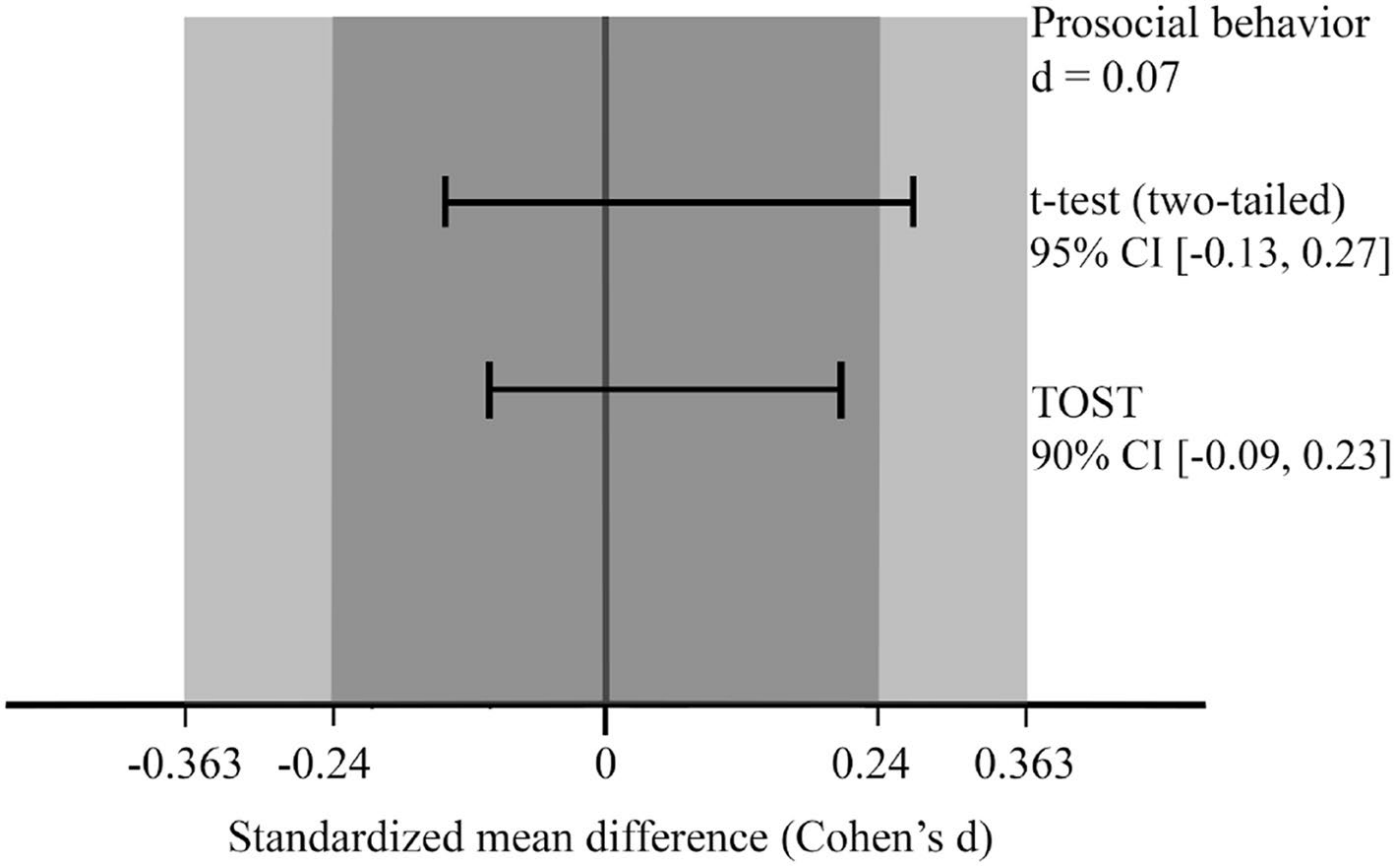
Standardized mean differences for the ‘Mask’ group and the ‘No Mask’ group in Study 1. *Note* The t-test indicates that the difference between the two groups is non-significantly different from 0. The TOST indicates that the difference between the two groups is significantly smaller than d = 0.363 and smaller than d = 0.24.

Given the non-significant results of the t-tests, we conducted an exploratory equivalence test on R with the TOSTER package^51^. While non-significant t-tests by themselves provide inconclusive null results, equivalence testing allows for a more informative interpretation of the null. This method consists in specifying a “null interval” or an equivalence region, which is an interval within which the effect size would be too small to be of any interest and is therefore negligible, and then in testing whether the observed effect is within the bounds of this interval of negligible effect sizes^52,53^. Testing whether an effect is within the bounds of a specified interval is conducted by the combination of two one-sided t-tests (TOST^54,55^), a first one to test whether the effect is greater than the lower bound of the null region and a second one to test whether the effect is smaller than the upper bound. The combination of the two t-tests conducted with an α err probability of 0.05 provides a 90% CI (i.e., 1–2α), and the result of the equivalence test is significant when the 90% CI is within the null region and does not overlap with any of the two bounds. The result for the TOST suggests that we can reject the presence of the initially hypothesized difference of d = 0.36 or larger, *t*(396) = 2.93, *p* = 0.002, 90% CI [− 0.097, 0.235] (see Fig. 2). Further exploration indicates that the p value for the TOST remains below 0.05 (*p* = 0.0496) for an effect as small as d = 0.24, 90% CI [− 0.097, 0.235].

### Discussion

The data from this first study did not support our predicted difference of d = 0.36 (12.8 questions) between the Mask and No Mask groups. There are two potential explanations for the null result. First, it may be that the lack of identifiability does not truly reduce prosocial behaviors. This would imply that conclusions in prior research in the literature were erroneous (i.e., false positives). Second, it may be that face masks do not actually reduce individuals’ perception of their own identifiability. One untested auxiliary assumption of our study was that individuals wearing sanitary face masks during the COVID-19 pandemic felt more anonymous and less identifiable, which is why, in turn, they would have behaved less prosocially. It is possible that, given that individuals focus on the eye area for face recognition^56^, individuals wearing face masks would expect others to be able to identify them even with the masks. We addressed these caveats in a second field study.

## Study 2

Having observed no significant difference in the first study, this time we hypothesized that participants wearing a mask and participants not wearing one would conduct equivalent levels of prosocial behaviors. We also measured perceived identifiability and hypothesized that individuals with and without a mask will have equivalent scores. Finally, we addressed the potential inflation of effect sizes in the literature by considering a much smaller effect size of interest. A recent study by Lovakov and Agadullina^57^ estimated that the median effect size for non-experimental studies in social psychology was d = 0.19. We rounded this value and considered an effect size of d = 0.2.

### Results

#### Descriptive statistics

From our N = 5308 participants, 2250 wore a mask during the entire interaction (i.e., Mask group) and 3058 did not (i.e., No Mask group).

The overall mean for the prosocial behavior measure was 8.38 with a standard deviation of 26.14, a minimum of 0 and a maximum of 106. The skewness of the distribution is 3.17 and the kurtosis is 8.58. As with the first study, in both groups, most participants answered 0 questions. The prosocial measure for the Mask group was M = 9.05 (SD = 27.06) and for the No Mask group was M = 7.88 (SD = 25.44).

The overall mean of the perceived identifiability measure was 3.12 (SD = 0.72), a minimum of 1.25 and a maximum of 5. The skewness of the distribution is 0.03 and the kurtosis is − 0.48. The perceived identifiability score for the Mask group was M = 3.05 (SD = 0.75) and for the No Mask group was M = 3.19 (SD = 0.68).

#### Hypothesis test

As per our pre-registration (https://doi.org/10.17605/OSF.IO/7642E), we conducted an equivalence test in the form of a TOST on R to test whether the number of questions answered by participants was equivalent in both groups. We defined equivalence by an effect size smaller than d = 0.2 (which corresponds to 5.22 questions answered out of 106). The result of the TOST was significant and indicates that we can reject the presence of a difference of d = 0.2 or larger, *t*(5306) = − 5.59, *p* < 0.001, 90% CI [− 0.0009, 0.09].

**Figure 3:**
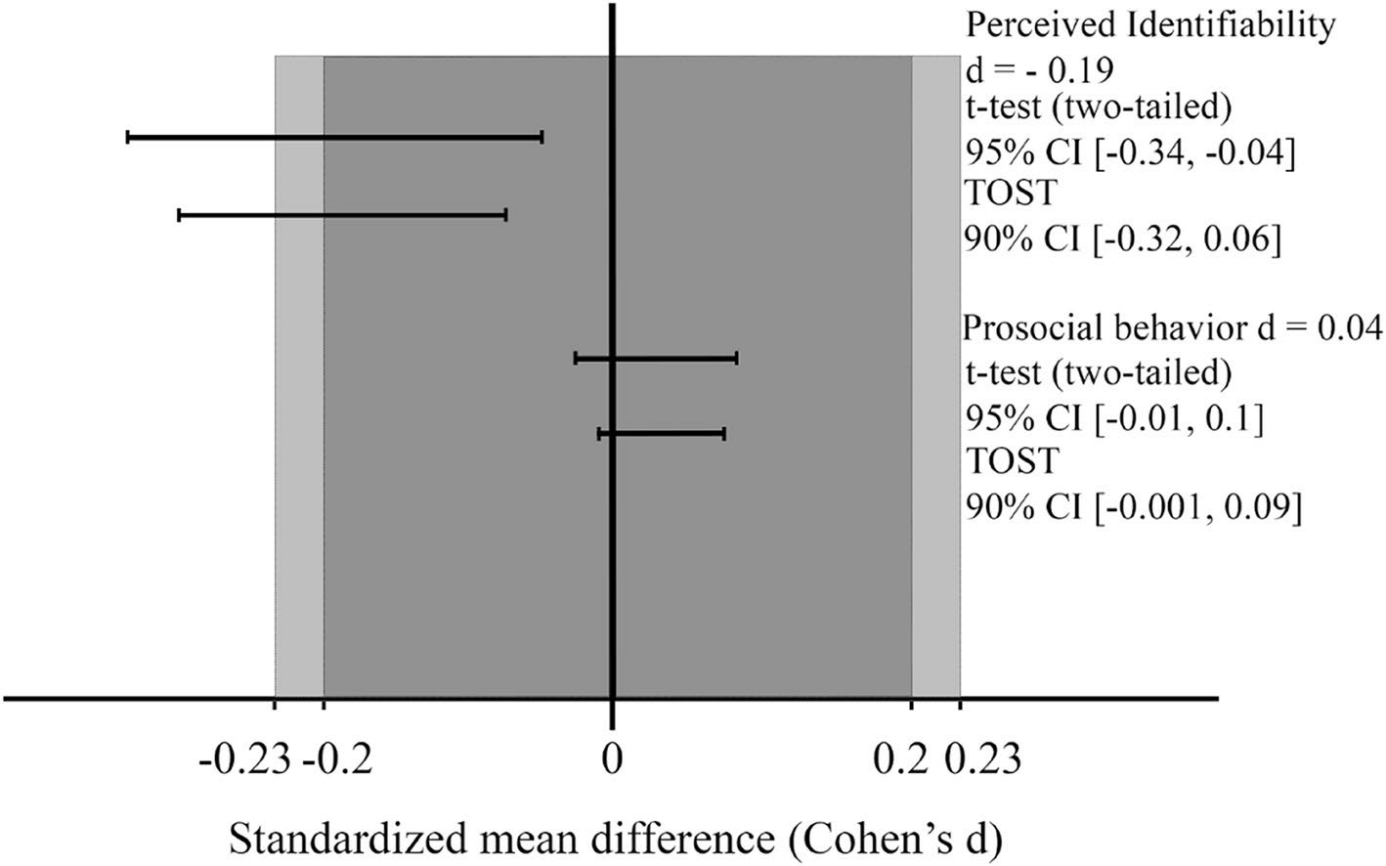
Standardized mean differences for the ‘Mask’ group and the ‘No Mask’ group in Study 2. *Note* The t-test for perceived identifiability indicates that the difference between the two groups is significantly different from 0. The TOST for perceived identifiability indicates that the difference between the two groups is not significantly smaller than d = 0.2, nor is it smaller than d = 0.23. The t-test for prosocial behavior indicates that the difference between the two groups is non-significantly different from 0. The TOST indicates that the difference between the two groups is significantly smaller than d = 0.2.

As per our pre-registration, we conducted a TOST to test whether the level of perceived identifiability was equivalent in both groups. Again, we defined equivalence by an effect size smaller than d = 0.2 (which corresponds to 0.143 point estimates). The result of the TOST was non-significant and indicates that we cannot reject the presence of a difference of d = 0.2 or larger, *t*(655) = 0.0524, *p* = 0.479, 90% CI [− 0.324, − 0.064]. (see Fig. 3).

Given our deviation from the pre-registration, we had a power of 64.16% to reject the presence of an effect of d = 0.2, but we had a power of 0.8 to reject the presence of an effect of d = 0.23, however, the result of the TOST was non-significant for a difference of d = 0.23 or larger either, *t*(655) = 0.437, *p* = 0.331, 90% CI [− 0.324, − 0.064].

#### Additional test

A non-significant TOST for the perceived identifiability measure only allows us to state that we cannot reject the presence of an effect of a pre-specified size. In order to test for the presence of an effect different from 0, we conducted an exploratory one-sided t-test to test whether perceived identifiability significantly decreased in the Mask group compared to the No Mask group. The difference between the group was significant, *t*(655) = − 2.414, *p* = 0.008, d = 0.19, 95% CI [− Infinity, − 0.04].

While the significant TOST for the prosocial behavior measure allows us to reject the presence of an effect of a pre-specified size, it does not allow the inference that the effect is non-zero. Thus, we conducted an exploratory one-sided t-test to test if the slight increase in prosocial behavior in the Mask group compared to the No Mask group was significantly different from zero, but this difference was not significant, *t*(5306) = − 1.62, *p* = 0.053, d = 0.04, 95% CI [− 0.01, Infinity].

We further explored to what degree we could narrow the null region of the equivalence test for the prosocial behavior and maintain a *p*-value below 0.05. The TOST remains significant (*p* = 0.023) for an effect as small as d = 0.01.

Among the 653 participants who answered the perceived identifiability measure, the level of perceived identifiability was not correlated to the measure of prosocial behavior, r = − 0.008, *p* = 0.83, 95% CI [− 0.08, 0.07]. We provide a wide range of alternative analyses in the supplementary materials.

Given the large number of participants answering zero questions, the results may have been impacted by a floor effect. Thus, we conducted the analysis by excluding participants who answered 0 questions. With the N = 670 participants who answered at least one question, the TOST would have been non-significant, t(668) = 0.375, *p* = 0.354, 90%CI [− 0.298, − 0.04]. Due to the large number of participants answering zero questions, we also conducted an analysis by dichotomizing responses between agreeing to answer (i.e., answering at least 1 question) and refusing to do so (i.e., answering 0 question). We conducted a logistic regression on the two-level categorical outcome. The results indicate that the odds of agreeing to answer the survey for the No Mask group was 0.13, 95% CI [0.11, 0.14], and the estimated odds ratio favored an increase of 1.32, 95% CI [1.12, 1.55] of agreeing to answer the survey for the Mask group, rather than a decrease.

### Discussion

Again, individuals wearing a face mask and individuals not wearing one had equivalent levels of prosocial behavior. Importantly, individuals wearing face masks do, in fact, have lower levels of perceived identifiability. This lower level of perceived identifiability, however, was not correlated to the level of prosocial behavior.

It is to be noted that one alternative analysis strategy would have favored an inconclusive result, while another would have favored the opposite hypothesis (i.e., that face masks increase prosocial behavior). Our main conclusion, however, relate to the pre-registered TOST with all participants, which, unlike the other analytic strategies, consider the full spectrum of responses from not being prosocial at all (i.e., refusing to answer any question) to being completely prosocial (i.e., answering all the questions) in the social interaction the participants had with our research assistants.

## General discussion

The present research showed that individuals wearing a face mask did not differ in terms of their level of willingness to take part in volunteer research from individuals without a mask, even though they had lower levels of perceived identifiability. The large sample size allows us to claim that face masks do not decrease prosocial behavior with almost no risk of type II error. These findings are inconsistent with previous research that found that reduced perceived identifiability results in reduced prosocial behavior. Although our study differs in many ways from previous recent studies such as Vesely and Klöckner^39^ and Zhong et al.^9^, it is noteworthy that these studies were considerably underpowered to detect typical social psychological effects. Recent meta-scientific work^57^ indicates that the median effect size in the field of social psychology overall is d = 0.36. The N = 136 by Vesely and Klöckner^39^ would have provided 67.13% power to detect an effect of d = 0.36. And the N = 50 and N = 83 for the study 2 and study 3 respectively by Zhong et al.^9^ would have provided only 23.87% and 36.73% power to detect such an effect. Underpowered studies may lead to largely overestimated effect sizes^58^ which would explain the effect sizes of d = 0.57 and d = 0.6 detected by Zhong et al.^9^. Given that our findings are based on high powered studies, we feel it more realistic to conclude at this point that perceived identifiability is unlikely to be related to prosocial behavior.

Some differences with previous research should be considered when relating our findings to the broader literature of deindividuation effects. It may be that the manipulations involved in other studies only reduce identifiability on the short term as they were conducted in laboratory conditions, whereas in our case individuals have been wearing face masks since the start of the pandemic in 2020 and has since become a habitual behavior. The earlier studies by Diener et al.^33^ and Miller and Rowold^34^ also focused on short term effects (i.e., being anonymous during the night of Halloween). It is thus possible that at the start of the pandemic, the social consequences of wearing a face masks were more salient and their impact on perceived identifiability were stronger than they are now. Recent work (e.g., Manley, Chan, and Wells^59^) show that identifying masked individuals is only sensibly more difficult than identifying unmasked individuals. If individuals are mostly confident in their ability to identify others who wear a mask, it then makes sense that face masks would have a modest effect on their own perceived identifiability.

Despite the differences between our studies and previous work, wearing a face mask in our study 2 was nonetheless associated with a decrease in perceived identifiability of d = − 0.19, but the level of perceived identifiability was not related to the level of prosocial behavior despite having 80% power to detect a correlation as small as r = − 0.09. It remains however possible that this decrease in perceived identifiability is not sufficient to impact prosocial behavior. The difference between the short-term effect of previous studies and the long-term usage of face mask may also imply that the effects found in the literature, should they indicate true effects, would dissipate over time.

When considering the implications of our results, one should also keep in mind that the design of our two studies does not allow us to test causal claims and there could be confounding variables that had an impact on the relationship between face masks and helping behavior. One possible limitation of our study may be that it was impacted by a selection bias. Participants that were wearing a mask might have been more altruistic in nature than those not wearing a mask, which then canceled out the effect of the first group being less identifiable (i.e., a potential decreased helping behavior). Similarly, unlike neutral objects such as sunglasses, face masks are themselves associated with prosociality (i.e., following national guidelines and protecting others) and may induce prosocial behavior which would counteract the hypothesized effect of reduced identifiability. Some previous research indeed suggest that clothing accessories may be related to behavior (see Adam and Galinsky^60^) including anti-normative behavior (e.g., Gino, Norton, and Ariely^61^). Another limitation was that our research assistants were always wearing face masks themselves. It is plausible that they were perceived as being part of an in-group with mask wearing participants which would, in turn, increase the level of prosocial behavior^62^ and thus undermined the potential negative effect of perceived identifiability on prosocial behavior. Note, however, that the measure of perceived identifiability was not related to the amount of helping in Study 2, suggesting it to be unlikely that wearing a face mask has a diminishing impact on prosocial behavior via decreased identifiability.

A final limitation of our conclusions concerns our measure of perceived identifiability itself. The explicit nature of the items may have induced our participants to focus their attention on the degree to which they are identifiable resulting in the observation that face masks decrease the level of perceived identifiability. It’s possible that if they are not explicitly asked about it, individuals wearing face masks would not necessarily feel less identifiable. However, given that neither our first study (without the perceived identifiability questions) nor our second study (with the perceived identifiability questions) provide evidence for an effect on prosocial behavior, our data provide no reason to believe that a potential question-behavior effect on perceived identifiability would impact prosocial behavior.

Regardless of these limitations, our two studies have strong significance from theoretical and practical perspectives. Taken together, our observations contribute to the cumulative knowledge in the prosocial literature and the deindividuation phenomenon as they provide strong evidence against the idea that perceived identifiability is related to prosocial behavior. Our findings support the conclusion of the meta-analysis by Postmes and Spears^37^ that there is little empirical support for a relation between deindividuation and anti-normative behavior. Although potential confounds could have influenced our findings, the null result, despite our statistical power of nearly 100%, points to the colossal sample sizes that would be required to conduct randomized controlled laboratory tests of these third variables. Nonetheless, our findings provide two positive implications. First, while our results show no decrease in self-interest behavior when wearing a face mask, we also provide strong evidence that individuals not wearing one do not behave more selfishly either. This, indirectly, points to the need to focus on alternative reasons why individuals don’t wear face masks. The second positive implication of our study should bring comfort to researchers in the field of psychological science. Notwithstanding the arduousness of finding participants for studies in ordinary times, researchers need not worry about additional potential hardship caused by face masks during the pandemic. As our results indicate, individuals agree to take part in studies all the same.

## Study 1 method

### Data collection and sampling strategy

We used a correlational observational design. Seven research assistants (five female and two male assistants) individually approached participants in public areas outside in Austria from the 19th of January 2021 to the 5th of February 2021 (average temperature = 1.59 °C, SD = 4.34). The research assistants presented themselves as university students who needed data to complete a course and earn credits. They were instructed to only approach solitary adults and to approach approximately the same number of individuals wearing a mask and not wearing a mask. The research assistants were always wearing a mask.

The sample size was determined a priori by a power analysis conducted on R Studio (R Studio Team, 2020) and the pwr package^63^ for a one-sided t-test, an α err probability of 0.05, a Power (1−β err probability) of 0.95 to observe an effect size of d = 0.363. We expected to observe an effect of this size by averaging the findings by Zhong et al.^9^, Kimmerle et al.^41^ and Cress and Kimmerle^40^. The study by Vesely and Klöckner^39^ was not considered for the power analysis because they confounded observability and identifiability. The minimum sample size, based on this power analysis was N = 330.

**Figure 4:**
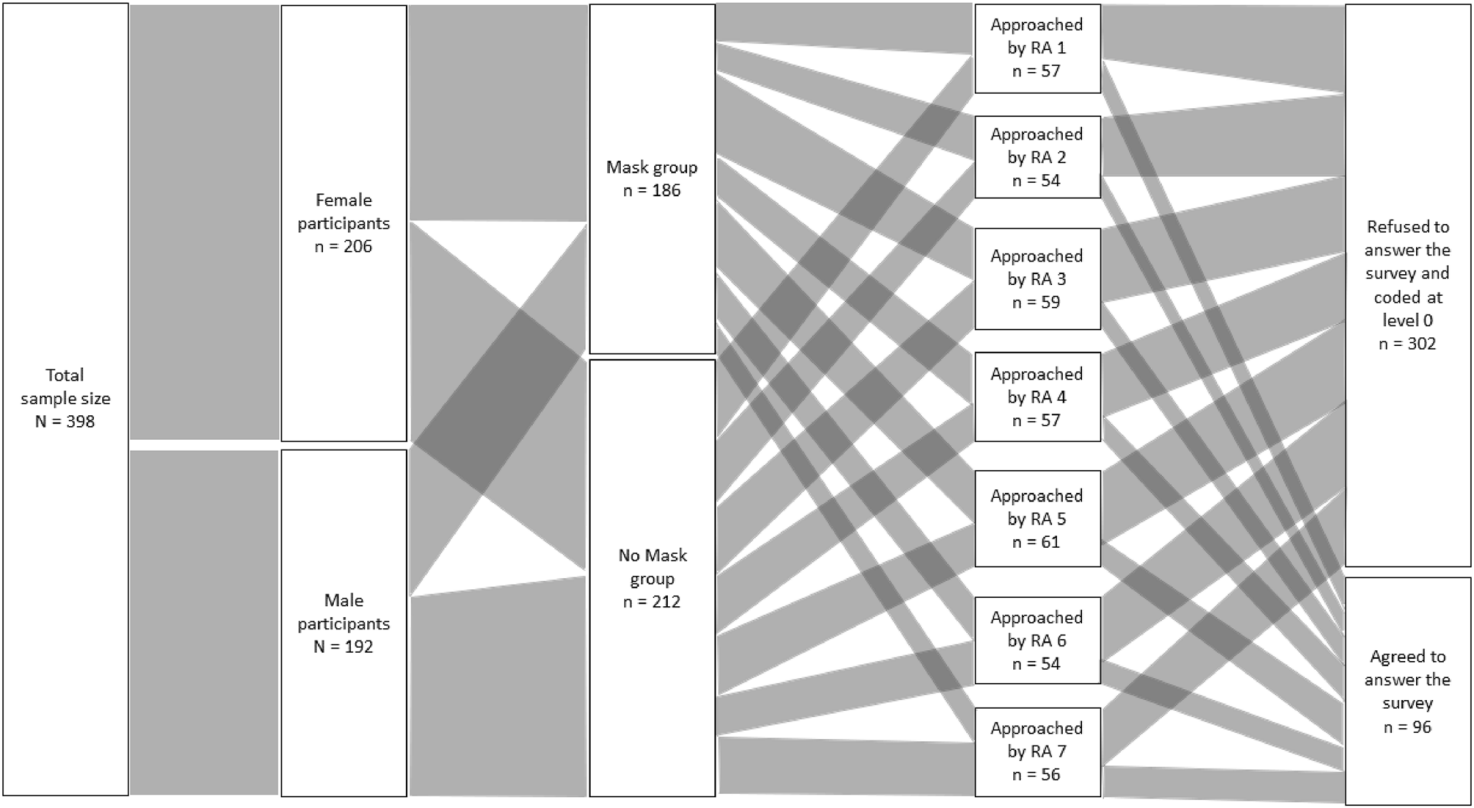
Alluvial diagram of the structure of our sample for study 1. *Note* This diagram represents the data collection process for the final sample we used in our main analysis. This diagram excludes participants who changed their mask status during the interaction with the research assistant. RA = Research Assistant.

A total number of 415 participants were approached. Our first two questions assessed age and occupation. All individuals who were approached and refused to answer any question were coded as having conducted the lowest level of prosocial behavior (see measures section), however, given that they did not answer any questions (including the first two) we could not assess the age and occupation for these participants (see Fig. 4). Age and occupation were only assessed for those who answered at least the first two questions, of which, four participants were below 18 years old and were excluded from the analysis. Thirteen participants changed their mask status during the interaction. In our main analysis, we also excluded these participants, but we also conducted alternative analyses by including these observations (see supplementary materials).

The final sample size consisted of N = 398 participants (206 females), of which n = 96 participants answered at least the first question (M_age_ = 36.79, SD_age_ = 16.50; 58 females). A sensitivity analysis showed that our actual sample size of N = 398 would have had a Power of 0.975 to detect the predicted effect of d = 0.363, and that the smallest effect size detectable with a Power of 0.95 would have been of d = 0.33.

### Measures

At the time of data collection, wearing a mask was mandatory inside closed public places (e.g., malls, shops, offices) but not outside, and participants were only approached outside. Therefore, all participants who wore a mask chose to do so.

To measure prosocial behavior, the research assistants asked participants to take part in a survey to help them earn credits for a university assignment. We used the German long version of the HEXACO survey^64^. The survey was selected for its length and because of the repetitive and monotonous aspect of the questions. Any other similar survey could have been used, but an existing standardized survey such as the HEXACO increases the realistic aspect of the study. The participants were given the survey in a 14-pages paper format and a pen. The research assistants emphasized that answering the questions was completely voluntary and that participants were free to answer the number of questions they wanted, however participants were informed that the more information they could provide the better. Participants were also reminded at the bottom of each page that they could stop if they wanted. Answering the questions was a costly behavior due to its length, monotonous aspect, and the discomfort of answering to 14-pages paper format survey while standing outside in the cold weather. But importantly, answering resulted in no material gain of any sort. Agreeing to do so was for the benefit of the research assistant, and the degree to which it would benefit the research assistant was determined by the number of questions. Prosocial behavior was then measured by the total number of questions the participants answered. Volunteering, being a form of prosocial behavior that may occur in individuals’ daily lives, is also related to other forms of prosocial behaviors such as cooperation (see Dovidio, Piliavin, Schroeder, and Penner^65^, p. 305).

The original survey had 100 questions and we included two questions about the age and occupation on the first page. Consequently, our measure of prosocial behavior ranged between 0 (answering no question) to 102 (answering all the questions). We chose to include participants who did not answer any question in order to assess the full range of the behavioral response, as not answering any question may be interpreted as not being prosocial at all in that situation and answering all the questions may be interpreted as being completely prosocial.

Given the predicted standardized effect size of d = 0.363, we predicted that participants in the Mask group would answer, on average, at least 12.8 fewer questions than participants in the No Mask group (i.e., 12.54% difference).

## Study 2 method

### Data collection and sampling strategy

The sampling procedure was the same as with the first study, with a few differences. This time we had four research assistants (2 male and 2 female assistants). The study was conducted from the 24th of March 2021 to the 18th of July 2021 (average temperature = 10.31, SD = 5.22).

As with our first study, we considered participants who refused to answer any question and they were coded as having conducted the lowest level of prosocial behavior. Consequently, and given that our measure of perceived identifiability was in the form of questions (see measures section), we had fewer participants for this latter measure than for the prosocial behavior measure (i.e., participants who did not answer the perceived identifiability questions were still counted in the prosocial behavior measure). For this reason, and in order to have enough power for both measures, we conducted an a priori power analysis on R Studio^66^ with the TOSTER package^51^ to estimate the minimum sample size to detect equivalence for the perceived identifiability measure only, and aimed to meet this number. With this approach, the sample size for the prosocial behavior would naturally be the same size or larger. As indicated in our pre-registration (https://doi.org/10.17605/OSF.IO/7642E), the minimum sample size we wished to obtain for the perceived identifiability measure was N = 858 which would have provided a Power (1-β err probability) of 0.80 to detect an effect of d = 0.2 should there be one. However, finding enough participants proved to be more complicated than we expected.

**Figure 5:**
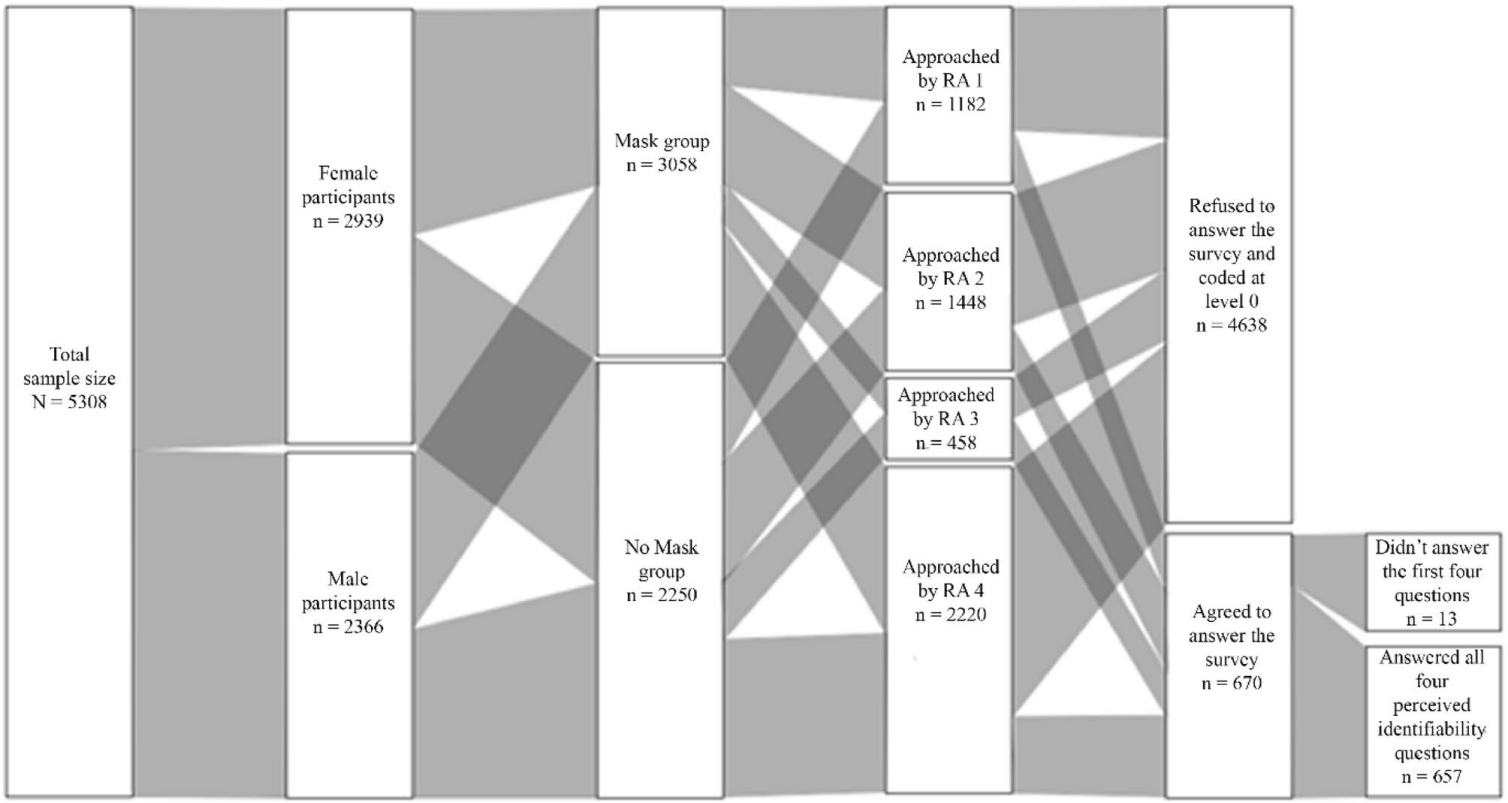
Alluvial diagram of the structure of our sample for study 2. *Note* This diagram represents the data collection process for the final sample we used in our main analysis. This diagram excludes participants who changed their mask status during the interaction with the research assistant. RA = Research Assistant.

For feasibility reasons, we had to deviate from the pre-registration and terminate the data collection with fewer participants after 133 days of data collection. After excluding 17 participants who were younger than 18 years and 85 participants who changed their mask status during the interaction, the sample size for the perceived identifiability measure consisted of N = 657 (n_Females_ = 385; M_age_ = 32.08, SD_age_ = 14.26), see Fig. 5. This sample provided a power of 80% to reject the presence of an effect size smaller than d = 0.23 (the power to reject the presence of an effect size smaller than d = 0.2 is then of 64.16%). The sample size for the prosocial behavior measure was N = 5308 (Females = 2939). This sample provided a power of nearly 100% to reject the presence of an effect size larger than d = 0.2, and the smallest detectable effect with a power of 0.80 was d = 0.076. It follows that we deviated from the pre-registration in regard to the sample size because the costs of collecting more data outweighed the benefits. Given that the effect size of d = 0.23 for the perceived identifiability measure is very close to our desired d = 0.2, and given the nearly 100% power for the prosocial behavior measure, we consider our study to provide informative observations nonetheless.

### Measures

Our addition to the method used in the first study was the measure of perceived identifiability. We included four items at the beginning of the survey that came after the questions regarding age and occupation. The questions were the German translation of the following:

1. “I feel anonymous right now.”
2. “I feel identifiable by others right now.”
3. “At this very moment I feel like nobody around me knows who I am.”
4. “If someone who knows me was walking near me at this very moment, they would be able to identify me.”

These four items were at the beginning to increase the likelihood that they would be answered by participants. In order to have a similar format as the HEXACO items, participants had to report on a 5-level scale to what level they agreed with each of the previous sentences (i.e., 1 = strongly disagree, 2 = disagree, 3 = neutral, 4 = agree, 5 = strongly agree). The items 2 and 4 and the reversed items 1 and 3 were averaged into an overall score of perceived identifiability. The four items had a Cronbach’s α of 0.61 and a McDonald’s ω of 0.66. Given the four additional questions, our measure of prosocial behavior ranged between 0 (answering no question) to 106 (answering all the questions).

## Supplementary Information

Supplementary Information.

## Data Availability

The datasets generated and analyzed during the current studies are available in the OSF repository: https://doi.org/10.17605/OSF.IO/CRZM3.
