# Supplementary material for: A practical test of the link between perceived identifiability and prosociality with two field studies
Source: Sci Rep. 2022 Jul 31;12:13149. doi: 10.1038/s41598-022-17248-2 (PMC9339540; doi:10.1038/s41598-022-17248-2)
Supplement: Supplementary file 1 — Supplementary Information. [file 41598_2022_17248_MOESM1_ESM.pdf]

STUDY 1 : Robustness checks

The overall mean for the prosocial measure did not significantly differ when comparing the data collected by each of the individual research assistants.

$F(6,391) = 1.061$, $p = 0.385$, $\eta p^2 = 0.02$, 95% CI [0.00, 0.03], $\omega 2 = 0.0009$, 95% CI [0.00, 0.00]

The result of our hypothesis test comparing the Mask and No Mask groups remained unchanged when excluding the data collected by each research assistant one by one.

When removing RA1: $t(339) = -0.763$, $p = 0.7769$ (one-tailed), $d = -0.08$, 95% CI [-0.26, Infinity]
When removing RA2: $t(342) = -0.331$, $p = 0.6297$ (one-tailed), $d = -0.04$, 95% CI [-0.22, Infinity]
When removing RA3: $t(337) = -0.388$, $p = 0.6508$ (one-tailed), $d = -0.04$, 95% CI [-0.23, Infinity]
When removing RA4: $t(339) = -0.975$, $p = 0.8349$ (one-tailed), $d = -0.11$, 95% CI [-0.28, Infinity]
When removing RA5: $t(335) = -0.325$, $p = 0.6272$ (one-tailed), $d = -0.04$, 95% CI [-0.21, Infinity]
When removing RA6: $t(342) = -0.386$, $p = 0.6503$ (one-tailed), $d = -0.04$, 95% CI [-0.22, Infinity]
When removing RA7: $t(340) = -1.252$, $p = 0.8943$ (one-tailed), $d = -0.14$, 95% CI [-0.32, Infinity])

The overall mean for the prosocial measure did not significantly differ when comparing the data collected by male research assistants vs. the female ones.

Mfemale = 18.63, SDfemale = 36.12, Mmale = 14.24, SDmale = 32.68, t(396) = 1.124, p =
0.262
(two-tailed), d = 0.12, 95% CI [-0.09, 0.34]

The conclusion of our hypothesis test remained unchanged when analyzing the data collected by male and female research assistants separately, although the direction of the effect is different.

Male RAs only: Mmask = 11.21, SDmask = 30.05, Mno mask = 17.21, SDno mask = 35.05,
t(111) =
0.975, p = 0.1659 (one-tailed), d = 0.18, 95% CI [-0.12, Infinity]

Female RAs only: Mmask = 21.9, SDmask = 37.79, Mno mask = 15.9, SDno mask = 34.55,
t(283) =
-1.399, p = 0.9187 (one-tailed), d= -0.17, 95% CI [-0.37, Infinity])

The overall mean for the prosocial measure differed significantly when comparing male and female participants.

Mmale = 13.45, SDmale = 31.01, Mfemale = 21.06, SDfemale = 38.39, t(396) = 2.165, p = 0.031
(two-tailed), d = 0.22, 95% CI [0.02, 0.41]

However, the conclusion of our hypothesis test remained unchanged when analyzing the data for male only or female only participants.

Male participants only: Mmask = 15.99, SDmask = 34.11, Mno mask = 11.63, SDno mask = 28.66,
t(190) = -0.958, p = 0.8304 (one-tailed), d = -0.14, 95% CI [-0.34, Infinity]

Female participants only: Mmask = 20.72, SDmask = 37.22, Mno mask = 21.42, SDno mask = 39.77,
t(204) = 0.131, p = 0.4479 (one-tailed), d = 0.02, 95% CI [-0.23, Infinity])

The overall mean for the prosocial measure did not significantly differ when comparing each combination of research assistant gender and participant gender.

$M_{male\ RA-male\ participant} = 10.02$, $SD_{male\ RA-male\ participant} = 26.92$, $M_{male\ RA-female\ participant} = 19.19$, $SD_{male\ RA-female\ participant} = 38.04$, $M_{female\ RA\ -\ male\ participant} = 15.05$, $SD_{female\ RA\ -\ male\ participant} = 32.75$, $M_{female\ RA\ -\ female\ participant} = 21.69$, $SD_{female\ RA\ -\ female\ participant} = 38.61$; $F(3,304) = 1.91$, $p = 0.127$, $\eta p^2 = 0.01$, 95% CI [0.00, 0.04], $\omega 2 = 0.006$, 95% CI [0.00, 0.02]

The result of our hypothesis test remained unchanged when excluding each of the combinations.

Without male RA-male participants: $M_{mask} = 15.58$, $SD_{mask} = 34.22$, $M_{no\ mask} = 13.92$, $SD_{no\ mask} = 31.4$, $t(242) = -0.394$, $p = 0.6532$ (one-tailed), $d = -0.05$, 95% CI [-0.24, Infinity]

Without male RA-female participant: $M_{mask} = 18.12$, $SD_{mask} = 35.22$, $M_{no\ mask} = 18.03$, $SD_{no\ mask} = 37.52$, $t(265) = 0.182$, $p = 0.4278$ (one-tailed), $d = 0.02$, 95% CI [-0.19, Infinity]

Without female RA-male participant: $M_{mask} = 19.52$, $SD_{mask} = 36.12$, $M_{no\ mask} = 15.17$, $SD_{no\ mask} = 33.66$, $t(344) = -1.156$, $p = 0.8759$ (one-tailed), $d = -0.13$, 95% CI [-0.3, Infinity]

Without female RA-female participant: $M_{mask} = 20.48$, $SD_{mask} = 37.29$, $M_{no\ mask} = 17.11$, $SD_{no\ mask} = 35.54$, $t(335) = -0.848$, $p = 0.8015$ (one-tailed), $d = -0.09$, 95% CI [-0.28, Infinity]).

The prosocial measure was not correlated to the average temperature during the data collection.

$t(396) = -0.212$, $p = 0.8321$, $r = -0.01$, 95% CI [-0.11, 0.09]

Among the participants who answered at least the first two questions (i.e., age and occupation on the first page), participant age was not correlated to the prosocial measure

$t(93) = 0.979$, $p = 0.3296$, $r = 0.1$, 95% CI [-0.1, 0.29]

Among the participants who answered at least the first two questions, the overall mean for the prosocial measure did not significantly differ when comparing those who were students ($M = 65.91$, $SD = 35.87$) and those who weren't students ($M = 74.4$, $SD = 34.15$; $t(93) = 1.01$, $p = 0.315$ (two-tailed), $d = 0.25$, 95% CI [-0.23, 0.72]).

And the result of our hypothesis test remained unchanged when excluding students.
$M_{mask} = 70.28$, $SD_{mask} = 34.76$, $M_{no\ mask} = 79.39$, $SD_{no\ mask} = 33.23$, $t(71) = 1.14$, $p = 0.1295$
(one-tailed), $d = 0.27$, 95% CI [-0.12, Infinity].

When excluding all the participants who did not answer any of the questions and were coded at the level 0 for the prosocial measure, the result of the hypothesis test remained unchanged.
Mmask = 69.5, SDmask = 35.51, Mno mask = 74.89, SDno mask = 33.61, t(94) = 0.76, p = 0.2239
(one-tailed), d = 0.16, 95% CI [-0.18, Infinity]

When excluding all the participants who answered all of the questions and were coded at thelevel 102 for the prosocial measure, the result of our hypothesis test also remained unchanged.
Mmask = 6.34, SDmask = 17.1, Mno mask = 4.26, SDno mask = 13.75, t(346) = -1.25, p = 0.8948
(one-tailed), d = -0.13, 95% CI [-0.14, Infinity]

When excluding the participants coded 0 and the participants coded 102 for the prosocial measure, the result of the hypothesis test also remained unchanged.
Mmask = 39.5, SDmask = 22.82, Mno mask = 39.65, SDno mask = 19.04, t(44) = 0.02, p = 0.4906
(one-tailed), d = 0.007, 95% CI [-0.3, Infinity]

Hence, it is unlikely that the “floor” and “ceiling” effects influence our conclusions.

When including participants who changed their mask status during the interaction by coding them according to how they were when they were approached (i.e., participants who had a mask but removed it were included in the Mask group and participants who didn't have a mask but put one on were included in the No Mask group), the results of the hypothesis test remained unchanged.

Mmask = 19.26, SDmask = 36.51, Mno mask = 17.2, SDno mask = 35.51, t(409) = -0.58, p = 0.7183
(one-tailed), d = -0.06, 95% CI [-0.23, Infinity]

When including these participants by coding them according to how they were at the end of the interaction (i.e., participants who put a mask on were included in the Mask group and participants who removed their mask were included in the No Mask group), the results of the hypothesis test also remained unchanged.

Mmask = 19.66, SDmask = 36.75, Mno mask = 16.82, SDno mask = 35.24, t(409) = -0.8, p = 0.7885
(one-tailed), d = -0.08, 95% CI [-0.25, Infinity]

When comparing the two groups with a Welch's t-test, the difference remains non-significant t(385.21) = -0.68, p = 0.7534 (one-tailed), d = -0.07, 95% CI [-0.23, Infinity])

When comparing the two groups with a Mann-Whitney U-test, the difference remains non-significant
U(Nmask = 186, Nno mask = 212) = 0.52322, z = 1.0714, p = 0.142 (one-tailed)

The participants 3, 9, 22, 28, 34, 35, 36, 61, 64, 65, 71, 72, 73, 127, 146, 148, 151, 152, 158, 162, 171, 181, 188, 192, 208, 226, 236, 238, 249, 257, 272, 278, 280, 281, 293, 294, 296, 298, 304, 305, 307, 308, 316, 340, 341, 347, 351, 386, 401, 403, 407 and 414 have been identified as having a Cook's distance greater than three times the mean Cook's distance of the sample for a model predicting the prosocial measure score by the Mask variable.

However, the conclusion of our hypothesis test remains unchanged when excluding these participants.

Mmask = 5.31, SDmask = 14.46, Mno mask = 4.26, SDno mask = 13.75, t(344) = -0.687, p = 0.7537
(one-tailed), d = -0.07, 95% CI [-0.1, Infinity]).

When analyzing the data according to whether participants agreed to help or not, regardless of the number of questions they answered, in the Mask group, 50 participants agreed to answer the survey, 136 refused, and in the No Mask group, 46 participants agreed to answer the survey, 166 refused.

With a logistic regression with a two-level categorical outcome variable, there is no significant difference between the Mask and No Mask group.
Unstandardized Bconstant = -1.28, SEconstant = 0.167, z = -7.7, $p < 0.001$; unstandardized Bmask =
0.28, SEmask = 0.23, z = 1.2, p =0.229).

The odds of agreeing to answer the survey for the No Mask group was 0.23, 95% CI [0.19, 0.38], and the estimated odds ratio favored an increase of 1.33, 95 % CI [0.84, 2.1] of agreeing to answer the survey for the Mask group.

STUDY 2 : Robustness checks

The overall mean for the prosocial measure significantly differed when comparing the data collected by each of the individual research assistants.

$F(3,5304) = 123.9$, $p < 0.001$, $\eta p^2 = 0.07$, 95% CI [0.05, 0.08], $\omega 2 = 0.06$, 95% CI [0.05, 0.08]

The result of our hypothesis test comparing the Mask and No Mask groups remained unchanged when excluding the data collected by each of the first three research assistants one by one.

When removing RA1: $t(4124) = -5.073$, $p < 0.001$ (TOST)

When removing RA2: $t(3858) = -5.932$, $p < 0.001$ (TOST)

When removing RA3: $t(4848) = -6.744$, $p < 0.001$ (TOST)

However, the result of our hypothesis test comparing Mask and No Mask groups differs when excluding the data collected by our fourth RA:

When removing RA4: $t(3086) = -1.299$, $p = 0.0971$ (TOST)

The overall mean for the prosocial measure significantly differed when comparing the data collected by male research assistants vs. the female ones.

$M_{female} = 17.44$, $SD_{female} = 36.45$, $M_{male} = 4.32$, $SD_{male} = 18.5$
$t(5306) = 17.36$, $p < 0.001$ (two-tailed), $d = 0.52$, 95% CI [0.46, 0.57]

The conclusion of our hypothesis test remained unchanged when analyzing the data collected by male and female research assistants separately, although the direction of the effect is different.

When removing male RAs: $t(1638) = 3.881$, $p < 0.001$ (TOST)

When removing female RAs: $t(3666) = 3.883$, $p < 0.001$ (TOST)

The overall mean for the prosocial measure differed significantly when comparing male and female participants.

$M_{male} = 7.26$, $SD_{male} = 24.29$, $M_{female} = 9.29$, $SD_{female} = 27.53$, $t(5303) = 2.8159$, $p = 0.004$
(two-tailed), $d = 0.08$, 95% CI [0.02, 0.13]

However, the conclusion of our hypothesis test remained unchanged when analyzing the data for male only or female only participants.

Male participants only: $t(2364) = 3.758$, $p < 0.001$ (TOST)

Female participants only: $t(2937) = -2.682$, $p = 0.003$ (TOST)

The overall mean for the prosocial measure significantly differed when comparing each combination of research assistant gender and participant gender.

$M_{male\ RA-male\ participant} = 4.48$, $SD_{male\ RA-male\ participant} = 18.88$, $M_{male\ RA-female\ participant} = 4.18$, $SD_{male\ RA-female\ participant} = 18.15$, $M_{female\ RA\ -\ male\ participant} = 15.33$, $SD_{female\ RA\ -\ male\ participant} = 34.41$, $M_{female\ RA\ -\ female\ participant} = 18.71$, $SD_{female\ RA\ -\ female\ participant} = 37.58$; $F(3,5301) = 103$, $p < 0.001$, $\eta p^2 = 0.06$,
95% CI [0.05, 0.06], $\omega 2 = 0.05$, 95% CI [0.04, 0.06]

The result of our hypothesis test remained unchanged when excluding each of the combinations.

Without male RA-male participants: $t(3542) = -3.715$, $p < 0.001$ (TOST)

Without male RA-female participant: $t(3397) = -4.665$, $p < 0.001$ (TOST)

Without female RA-male participant: $t(4698) = -5.092$, $p < 0.001$ (TOST)

Without female RA-female participant: $t(4270) = 5.712$, $p < 0.001$ (TOST)

The prosocial measure was not correlated to the average temperature during the data collection.

$t(5306) = -0.5309$, $p = 0.5955$, $r = -0.007$, 95% CI [-0.03, 0.02]

Among the participants who answered at least the first two questions (i.e., age and occupation on the first page), participant age was not correlated to the prosocial measure

$t(655) = 1.7549$, $p = 0.0797$, $r = 0.068$, 95% CI [-0.008, 0.14]

Among the participants who answered at least the first two questions, the overall mean for the prosocial measure did not significantly differ when comparing those who were students ($M = 62.98$, $SD = 39.76$) and those who weren't students ($M = 69.03$, $SD = 38.93$; $t(650) = 1.9307$, $p = 0.05395$ (two-tailed), $d = 0.15$, 95% CI [0, 0.31]).

And the result of our hypothesis test remained unchanged when excluding students.
$t(5042) = -6.107$, $p < 0.001$ (TOST)

Among the participants who answered at least the first two questions, the overall mean for the prosocial measure did not significantly differ when comparing those who had an activity related to psychology ($M = 72$, $SD = 32.74$) and those who didn’t have an activity related to psychology ($M = 66.53$, $SD = 39.42$; $t(650) = -0.33846$, $p = 0.7351$ (two-tailed), $d = -0.14$, 95% CI [-0.94, 0.67]).

And the result of our hypothesis test remained unchanged when excluding those who had an activity related to psychology.
$t(5300) = -5.525$, $p < 0.001$ (TOST)

When excluding all the participants who did not answer any of the questions and were coded at the level 0 for the prosocial measure, the result of the hypothesis test was non-significant.

$t(668) = 0.375$, $p = 0.354$ (TOST)

When excluding all the participants who answered all of the questions and were coded at the level 106 for the prosocial measure, the result of our hypothesis test remained unchanged.

$t(5022) = -5.783$, $p < 0.001$ (TOST)

When excluding the participants coded 0 and the participants coded 106 for the prosocial measure, the result of the hypothesis test was non-significant.

$t(384) = -0.435$, $p = 0.668$ (TOST)

Hence, there is a possibility that the “floor effect” has impacted our results.

When including participants who changed their mask status during the interaction by coding them according to how they were when they were approached (i.e., participants who had a mask but removed it were included in the Mask group and participants who didn’t have a mask but put one on were included in the No Mask group), the results of the hypothesis test remained unchanged.

$t(5408) = 6.957$, $p < 0.001$ (TOST)

When including these participants by coding them according to how they were at the end of the interaction (i.e., participants who put a mask on were included in the Mask group and participants who removed their mask were included in the No Mask group), the results of the hypothesis test also remained unchanged.

$t(5408) = 3.861$, $p < 0.001$ (TOST)

The following participants have been identified as having a Cook's distance greater than three times the mean Cook's distance of the sample for a model predicting the prosocial measure score by the Mask variable:

4, 104, 105, 141, 159, 160, 164, 174, 205, 209, 217, 220, 241, 249, 252, 313, 329, 349, 432, 453, 468, 512, 531, 545, 551, 553, 557, 571, 606, 624, 630, 648, 682, 691, 731, 777, 845, 922, 1000, 1527, 1560, 2018, 2025, 2028, 2033, 2037, 2038, 2043, 2055, 2058, 2063, 2071, 2083, 2092, 2097, 2103, 2110, 2122, 2126, 2144, 2157, 2174, 2187, 2200, 2216, 2218, 2220, 2251, 2253, 2276, 2278, 2294, 2303, 2312, 2318, 2323, 2328, 2342, 2349, 2398, 2401, 2410, 2416, 2428, 2438, 2468, 2470, 2473, 2490, 2491, 2500, 2517, 2518, 2519, 2524, 2538, 2546, 2549, 2571, 2577, 2602, 2610, 2613, 2614, 2631, 2644, 2659, 2661, 2664, 2668, 2684, 2697, 2699, 2700, 2716, 2717, 2723, 2724, 2729, 2731, 2739, 2741, 2742, 2752, 2754, 2756, 2765, 2775, 2782, 2788, 2797, 2801, 2821, 2822, 2830, 2835, 2858, 2867, 2873, 2875, 2876, 2885, 2893, 2898, 2904, 2914, 2917, 2919, 2942, 2947, 2953, 2960, 2961, 2962, 2967, 3021, 3036, 3059, 3065, 3074, 3091, 3098, 3120, 3145, 3146, 3170, 3188, 3203, 3245, 3249, 3271, 3287, 3293, 3309, 3341, 3361, 3380, 3394, 3397, 3409, 3431, 3442, 3459, 3478, 3486, 3504, 3514, 3529, 3545, 3577, 3649, 3683, 3724, 3732, 3743, 3767, 3775, 3788, 3796, 3815, 3831, 3843, 3859, 3903, 3922, 3932, 3939, 3956, 3962, 3976, 3998, 4006, 4020, 4050, 4063, 4070, 4078, 4091, 4097, 4100, 4101, 4116, 4124, 4129, 4137, 4140, 4142, 4147, 4148, 4155, 4164, 4170, 4182, 4191, 4193, 4205, 4207, 4211, 4213, 4217, 4218, 4220, 4225, 4226, 4233, 4235, 4239, 4240, 4242, 4246, 4247, 4250, 4251, 4257, 4258, 4263, 4268, 4271, 4272, 4273, 4279, 4282, 4283, 4284, 4285, 4286, 4287, 4288, 4289, 4290, 4291, 4293, 4294, 4295, 4296, 4297, 4298, 4299, 4300, 4312, 4331, 4332, 4337, 4342, 4343, 4346, 4347, 4361, 4365, 4372, 4376, 4377, 4378, 4379, 4405, 4411, 4416, 4417, 4661, 4669, 4675, 4680, 4688, 4700, 4713, 4723, 4725, 4729, 4735, 4739, 4740, 4750, 4759, 4767, 4772, 4776, 4781, 4789, 4791, 4799, 4802, 4817, 4830, 4837, 4860, 4871, 4917, 4941, 4942, 4966, 4982, 5004, 5010, 5028, 5047, 5056, 5066, 5067, 5089, 5115, 5134, 5142, 5176, 5181, 5182, 5185, 5186, 5202, 5204, 5205, 5213, 5223, 5224, 5226, 5235, 5236, 5241, 5243, 5250, 5252, 5253, 5258, 5262, 5263, 5270, 5274, 5302, 5308, 5315, 5321, 5337, 5340, 5346, 5356, 5385, 5398, 5402, 5409, 5410.

However, the conclusion of our hypothesis test remains unchanged when excluding these participants.

$t(4927) = 6.578$, $p < 0.001$ (TOST)

When analyzing the data according to whether participants agreed to help or not, regardless of the number of questions they answered, in the Mask group, 322 participants agreed to answer the survey, 1925 refused, and in the No Mask group, 344 participants agreed to answer the survey, 2709 refused.

With a logistic regression with a two-level categorical outcome variable, there is no significant difference between the Mask and No Mask group.

Unstandardized Bconstant = -2.06, SEconstant = 0.06, $z = -36.07$, $p < 0.001$; unstandardized Bmask =
0.28, SEmask = 0.08, $z = 3.34$, $p < 0.001$).

The odds of agreeing to answer the survey for the No Mask group was 0.13, 95% CI [0.11, 0.14], and the estimated odds ratio favored an increase of 1.32, 95 % CI [1.12, 1.55] of agreeing to answer the survey for the Mask group.

The overall mean for the perceived identifiability measure significantly differed when comparing the data collected by each of the individual research assistants.

$F(3,653) = 4.467$, $p = 0.004$, $\eta p^2 = 0.02$, 95% CI [0.00, 0.04], $\omega 2 = 0.02$, 95% CI [0.00, 0.03]

The result of our hypothesis test comparing the Mask and No Mask groups remained unchanged when excluding the data collected by each research assistant one by one.

When removing RA1: $t(439) =-0.654$, $p = 0.743$ (TOST)

When removing RA2: $t(538) = -0.856$, $p = 0.804$ (TOST)

When removing RA3: $t(498) = -2.425$, $p = 0.583$ (TOST)

However, the result of our hypothesis test comparing Mask and No Mask groups differs when excluding the data collected by our fourth RA:

When removing RA4: $t(488) = 1.898$, $p = 0.0292$ (TOST)

The overall mean for the perceived identifiability measure did not significantly differ when comparing the data collected by male research assistants vs. the female ones.

$M_{female} = 3.14$, $SD_{female} = 0.68$, $M_{male} = 3.09$, $SD_{male} = 0.77$, $t(655) = 0.886$, $p = 0.37$ (two-tailed),
$d = 0.07$, 95% CI [-0.08, 0.22]

The conclusion of our hypothesis test remained unchanged when analyzing the data collected by male and female research assistants separately, although the direction of the effect is different.

When removing male RAs: $t(371) = 1.211$, $p = 0.113$ (TOST)

When removing female RAs: $t(282) = -1.398$, $p = 0.918$ (TOST)

The overall mean for the perceived identifiability measure did not significantly differ when comparing male and female participants.

$M_{male} = 3.08$, $SD_{male} = 0.7$, $M_{female} = 3.15$, $SD_{female} = 0.73$, $t(655) = 1.172$, $p = 0.2413$ (two-tailed), $d = 0.09$, 95% CI [-0.06, 0.25]

The conclusion of our hypothesis test remained unchanged when analyzing the data for male only or female only participants.

Male participants only: $t(270) = -0.262$, $p = 0.603$ (TOST)

Female participants only: $t(383) = 0.340$, $p = 0.367$ (TOST)

The overall mean for the perceived identifiability measure did not significantly differ when comparing each combination of research assistant gender and participant gender. $M_{male\ RA\text{-}male\ participant}$ = 3.08, $SD_{male\ RA\text{-}male\ participant}$ = 0.74, $M_{male\ RA\text{-}female\ participant}$ = 3.11, $SD_{male\ RA\text{-}female\ participant}$ = 0.8, $M_{female\ RA\ -\ male\ participant}$ = 3.08, $SD_{female\ RA\ -\ male\ participant}$ = 0.64, $M_{female\ RA\ -\ female\ participant}$ = 3.17, $SD_{female\ RA\ -\ female\ participant}$ = 0.69; $F(3,653) = 0.721$, $p = 0.539$, $\eta p^2 = 0.0033$, 95% CI [0.00, 0.01], $\omega 2 = -0.0013$, 95% CI [0.00, 0.00]

The result of our hypothesis test remained unchanged when excluding each of the combinations.

Without male RA-male participants: $t(510) = 1.149$, $p = 0.126$ (TOST)

Without male RA-female participant: $t(516) = 0.153$, $p = 0.439$ (TOST)

Without female RA-male participant: $t(528) = -0.696$, $p = 0.757$ (TOST)

Without female RA-female participant: $t(409) = -0.346$, $p = 0.635$ (TOST)

The perceived identifiability measure was not correlated to the average temperature during the data collection.

$t(655) = -0.821$, $p = 0.4118$, $r = -0.032$, 95% CI [-0.1, 0.04]

Among the participants who answered at least the first two questions (i.e., age and occupation on the first page), participant age was not correlated to the perceived identifiability measure

$t(646) = 1.6077$, $p = 0.1084$, $r = 0.063$, 95% CI [-0.01, 0.14]

The overall mean for the perceived identifiability measure did not significantly differ when comparing those who were students ($M = 3.09$, $SD = 0.74$) and those who weren't students ($M = 3.15$, $SD = 0.7$; $t(642) = 0.94434$, $p = 0.3454$ (two-tailed), $d = 0.08$, 95% CI [-0.08, 0.23]).

And the result of our hypothesis test remained unchanged when excluding students.

$t(395) = -0.0034$, $p = 0.501$ (TOST)

The overall mean for the perceived identifiability measure did not significantly differ when comparing those who had an activity related to psychology ($M = 3.25$, $SD = 0.52$) and those who didn't ($M = 3.12$, $SD = 0.72$; $t(642) = -0.431$, $p = 0.666$ (two-tailed), $d = -0.18$, 95% CI [-0.98, 0.63]).

And the result of our hypothesis test remained unchanged when excluding those who had an activity related to psychology.

$t(649) = 0.0525$, $p = 0.479$ (TOST)

The following participants have been identified as having a Cook's distance greater than three times the mean Cook's distance of the sample for a model predicting the perceived identifiability measure score by the Mask variable:

174, 624, 682, 1075, 1125, 1153, 1221, 1323, 1487, 1533, 1757, 1809, 1910, 2097, 2170, 2438, 2445, 2473, 2668, 2692, 2694, 2746, 2752, 2858, 2917, 2942, 2953, 2961, 3000, 3271, 3545, 3563, 3815, 4053, 4070, 4193, 4250, 4332, 4337, 4405, 4781, 5066, 5110, 5204, 5213, 5235, 5341, 5398.

However, the conclusion of our hypothesis test remains unchanged when excluding these participants.

$t(607) = 0.861$, $p = 0.195$ (TOST)

When computing the score of perceived identifiability by excluding each item one by one, the result of our hypothesis test remains unchanged.

When removing item 1: $t(655) = 0.273$, $p = 0.392$ (TOST)

When removing item 2: $t(657) = 0.294$, $p = 0.384$ (TOST)

When removing item 3: $t(655) = -0.098$, $p = 0.539$ (TOST)

When removing item 4: $t(655) = 0.527$, $p = 0.99$ (TOST)
